# Mind-Brain Dualism in Psychiatry: Ethical Implications

**DOI:** 10.3389/fpsyt.2020.00085

**Published:** 2020-03-03

**Authors:** Walter Glannon

**Affiliations:** Philosophy, University of Calgary, Calgary, AB, Canada

**Keywords:** biological psychiatry, brain-mind interaction, dualism, global burden of disease, major psychiatric disorders, neuromodulation

## Introduction

Psychiatric disorders are often described as disorders of the mind. Major depressive disorder (MDD), generalized anxiety disorder (GAD), obsessive-compulsive disorder (OCD), and posttraumatic stress disorder (PTSD) are categorized by varying degrees of psychomotor, cognitive, affective, and volitional impairment (1). Many explain them in psychological terms without referring to an underlying neural substrate (2). This position may be traced to Freud's failed attempt to link neural mechanisms to psychodynamic concepts in his *Project for a Scientific Psychology*. It led him to abandon neurology in favor of psychoanalysis (3). Karl Jaspers later stated that biological and psychological investigations of the mind are like “the exploration of an unknown continent from opposite directions, where the explorers never meet because of the impenetrable country that intervenes (4).” Jaspers was not endorsing substance dualism, the theory that brain and mind are ontologically distinct material and immaterial substances (5). He was making an epistemological claim, noting that we have an incomplete understanding of the brain and mind and how they interact. Some contemporary psychiatrists seem to interpret the idea of biology and psychology coming from “opposite directions” as suggesting an epistemological and explanatory dualism between neural and mental processes. This appears to be part of an “identity crisis” in psychiatry reflecting disagreement about characterizing psychiatric disorders as disorders of the mind or brain (6). Dualism as such does not preclude mind-brain interaction. But it supports the position that mind and brain can be functionally distinct. I argue that this is not consistent with neuroscience research showing the extent to which mental and neural processes are interdependent and influence each other in maintaining mental health or causing mental illness. Dualistic thinking of the type I have described can limit therapeutic interventions for patients suffering from major psychiatric disorders.

## Mind and Brain

Research in clinical neuroscience can be interpreted to imply that there is no impenetrable barrier between mind and brain in psychiatry. Major psychiatric disorders are not just of the mind *or* brain, but of the mind *and* brain. This rejection of dualism has significant ethical implications. A unified model explaining the extent to which mental and neural processes interact could lead to safer and more effective treatments to control and ideally prevent psychiatric disorders. This could maximize benefit and minimize harm to the millions of people suffering from them for the balance of their lives. It could provide a theoretical and clinical basis for psychiatrists to discharge their obligations of beneficence and nonmaleficence in treating patients (7). It could also disabuse many of the idea that mental illness is all in the mind and completely within our conscious control to avoid or resolve. This could prevent affected people from feeling responsible for their illness and thus prevent additional psychological harm. We cannot explain mental processes apart from neural processes, or vice versa. There is no mind without brain and no brain without mind (8). They are functionally interdependent. Normal mind-brain interaction enables persons to adapt to the world. In major psychiatric disorders, there is dysfunction at both mental and neural levels. Indeed, an adequate explanatory model for these disorders, as well as for interventions to treat them, requires an account of not just interaction between the mind and brain, but also how genetic, epigenetic, endocrine, immune and environmental factors influence this interaction.

Nonreductive materialism may provide a satisfactory theory of mind-brain interaction in psychiatry (9). The brain necessarily generates and sustains mental events and processes (10). But these are not reducible or identical to neural events and processes. Mental phenomena are partly but not completely explained in terms of their neural correlates (11). As the comments and examples in the next two sections illustrate, this position rejects the view that mental states are epiphenomenal and cannot cause changes in the brain (12, 13). Consistent with nonreductive materialism, neurobiological naturalism explains mind and brain as interdependent components of a human organism. The mind emerges from the brain when it reaches a certain level of organization and complexity (14). Neural and mental functions constrain each other in a nested hierarchy of reentrant loops that maintain homeostasis in the organism and promote adaptability to the environment (15). The subjectivity and intentionality of mental states provide a person with a more accurate representation of the world than the representation provided by the brain alone (16). Mutual neural and mental constraint prevents misrepresentation of the world, as in psychoses, and hyperactive responses to aversive stimuli, as in stress-induced anxiety and depression. Major psychiatric disorders develop when something goes awry in these processes. The idea that mind and brain are functionally interdependent rather than functionally independent systems was accepted by many neurologists in the nineteenth century. They included Paul Broca, who claimed that “the great regions of the mind correspond to the great regions of the brain (17).” He was not making a reductionist claim but emphasizing how mind and brain have complementary roles in maintaining motor and mental functions.

## Biological Psychiatry

According to one definition of biological psychiatry, “mental disorders are relatively stable, prototypical dysfunctional patterns of experience and behavior that can be explained by dysfunctional systems at different levels (18–20).” The systems on which this field has focused are dysfunctional neurotransmitters and neural circuits in cortical and subcortical regions of the brain and how they generate different types and degrees of mental impairment. Because of increased knowledge of the function of the excitatory neurotransmitter glutamate, studies have shown that intranasal delivery of the noncompetitive NMDAR antagonist esketamine can have rapid therapeutic effects in some people with treatment-resistant depression (21). This is significant because the pharmaceutical industry has largely left a therapeutic vacuum in psychiatry by substantially reducing its investment in the development of new psychotropic drugs. Deep brain stimulation (DBS) and other forms of neuromodulation can ameliorate symptoms in some patients with treatment-resistant MDD and OCD (22, 23). Genome-wide association studies can help to identify people at risk of developing these and other psychiatric disorders (24). In addition, the identification of biomarkers with functional neuroimaging has clarified why some individuals with depression respond or fail to respond to antidepressants or psychotherapy (25). The Research Domain Criteria (RDoC) is grounded in biological psychiatry (26). Unlike the symptom-based DSM-5, the aim of the RDoC is to identify abnormal brain mechanisms that can explain the etiology and pathophysiology of psychiatric disorders and provide earlier and more accurate diagnosis to produce optimal responses and outcomes (27–29).

Biological psychiatry does not exclude psychology. As Henrik Walter points out, “many proponents of biological psychiatry now accept an interplay of neurological and psychological (mental) factors” in explaining psychiatric disorders (18). Therapies based on this interplay can relieve or control symptoms of these disorders more effectively than therapies targeting mental or neural processes alone. Broadly construed, biological psychiatry is based on interaction between brain, mind, body and environment. Even with this broad scope, it has not generated a complete understanding of this interaction and can only approximate this goal with continued research.

## Mental-Neural Interaction

Trauma or chronic psychosocial stress can disrupt neural mechanisms maintaining normal mental functions. A hyperactive psychological response to aversive stimuli can trigger a cascade of neurophysiological events causing dysregulation of the hypothalamic-pituitary-adrenal (HPA) axis and result in the symptoms of MDD or GAD (30). They do not begin as brain disorders but become brain disorders over time. The deleterious neural and mental effects of high circulating levels of cortisol from the adrenal cortex and norepinephrine from the adrenal medulla through the locus coeruleus to the amygdala show that mind and brain interact not only with the environment but also the endocrine system. In addition, the role of cytokines in depression is an example of how the immune system can affect the central nervous system (31). The mind can have positive effects in the brain as well. Cognitive behavioral therapy (CBT) can rewire cortico-limbic pathways, resulting in improved cognition and mood for some patients with depression (32, 33). Neurofeedback using EEG or fMRI is another example of how psychological responses to brain activity can regulate it. The use of this technique to improve mood and motivation in depressed patients is an example of “a holistic approach that overcomes bio-psychological dualisms” (34).

Some authors cataloguing the history of psychiatry end their analyses by emphasizing the limits of psychopharmacology (35). They fail to consider how neuromodulation and psychological therapies may be part of a comprehensive treatment plan for moderately severe to severe psychiatric disorders. As noted, major psychiatric disorders involve not only dysfunctional neurotransmitters but also dysfunctional neural circuits (36). DBS can modulate a dysfunctional fronto-striatal circuit in OCD enough to make it amenable to CBT or other behavioral or psychotherapeutic techniques (37). Combining therapies targeting both neural and mental processing may enable patients to unlearn maladaptive thought and behavior. Focusing only on the mind or brain and failing to appreciate how each influences the other could preclude complementary treatment modalities to improve response rates and relieve symptoms. They could modulate hyperactive or hypoactive brain-mind processing to restore homeostasis and flexible action. By applying this knowledge of neural and mental interaction in research and practice, psychiatrists can more effectively discharge their obligations of nonmaleficence and beneficence to research subjects and patients.

Epigenetic factors influencing gene expression in the brain can shape an individual's response to psychosocial stress. “Growing evidence supports the hypothesis that epigenetics is a key mechanism through which environmental exposures interact with an individual's constitution and influence gene expression to determine risk for depression throughout life (38).” Research could identify epigenetic changes caused by environmental stressors that could influence individuals' susceptibility or resilience to depression. Altering the natural and social environment to reduce external stressors could reduce the risk of developing this disorder. In addition, an integrated model explaining how genetic, epigenetic and environmental factors can dysregulate fear conditioning in PTSD might be able to predict which environments would be more likely to cause the disorder and how it might be prevented (39).

In psychoses, the impaired ability of anterior cortical brain regions to inhibit dysregulated posterior cortical and subcortical regions can result in auditory or visual hallucinations, delusions and other abnormal conscious states. Genetic and neurobiological mechanisms alone seem to account for them. Structural imaging showing gray and white matter abnormalities and functional imaging showing dysfunctional cortico-striatal connectivity in the brains of people with the positive subtype of schizophrenia confirm that they are diseases of the brain with symptoms in distorted mental content (40). This does not mean that the mind cannot have a therapeutic role in this or other psychotic disorders. Studies have shown that a combination of antipsychotic medication, psychotherapy, family support and continued work and social activity results in improved cognitive, affective and volitional function and greater independence among adolescents when initiated shortly after a first-episode psychosis (41, 42). This biopsychosocial approach to treating schizophrenia is another example of how rejecting dualistic mind-or-brain models can increase benefit and reduce harm in people with major psychiatric disorders. Biological psychiatry does not imply that ordered and disordered mental states can be explained entirely in terms of ordered and disordered brain processes. But it does imply that psychomotor, cognitive, affective, and volitional dysfunction correlates with dysfunctional neural networks and can be treated by modulating these networks through neurobiological and psychological interventions.

## Conclusion

Caleb Gardner and Arthur Kleinman claim that “biological psychiatry has thus far failed to produce a comprehensive theoretical model of any major psychiatric disorder (6)…” While their comment draws attention to the limitations of biological psychiatry, it does not discredit it or indicate that it should be replaced by a psychological or social research model that excludes neurobiology. Instead, it underscores that it is a work in progress and the need for more research to explain the complex interaction between neural and psychological processes in mental health and illness. Gardner and Kleinman add that “In many ways, the unknown continent of the mind looms even larger now than it did in Jaspers' day—a reality that is both humbling and inspiring (6).” The first part of this comment suggests dualistic thinking about the mind as a mysterious domain epistemologically and explanatorily distinct from the brain. It contributes to the idea that we need to choose between characterizing psychiatric disorders as disorders of the brain or mind. This is a false dichotomy given that brain-mind and mind-brain interaction enables or disables thought and behavior. Yet the second part of their comment points to the motivation for research that will provide a better understanding of how brain and mind influence each other.

“What the research of the past decades has shown us most convincingly is that biology and environment work powerfully together on the brain and the mind—and that psychiatry has hit its roadblock because we know too little about how the brain functions (43).” Biological psychiatry is not part of this roadblock. While much more work is needed, it has the potential to yield a better understanding of major psychiatric disorders by generating increased knowledge of neurobiological, psychological and environmental interaction needed to predict, treat, and prevent them. This is significant because psychiatric disorders constitute a higher percentage of the global burden of disease than cancer and other diseases (44). Research that can generate this knowledge may clarify the etiology and pathophysiology of these disorders. This may advance interventions enabling functional independence and improved quality of life for the millions of people affected by them. Taking the full extent of mind-brain interaction into account is thus ethically imperative in psychiatric research and practice.

## Author Contributions

The author confirms being the sole contributor of this work and has approved it for publication.

## Conflict of Interest

The author declares that the research was conducted in the absence of any commercial or financial relationships that could be construed as a potential conflict of interest.
